# A structural equation mediation model captures the predictions amongst the parameters of the ease of language understanding model

**DOI:** 10.3389/fpsyg.2023.1015227

**Published:** 2023-03-03

**Authors:** Lina Homman, Henrik Danielsson, Jerker Rönnberg

**Affiliations:** ^1^Disability Research Division (FuSa), Department of Behavioural Sciences and Learning (IBL), Linköping University, Linköping, Sweden; ^2^Linnaeus Centre HEAD, The Swedish Institute for Disability Research, Linköping University, Linköping, Sweden

**Keywords:** ELU model, working memory, mediation model, cognitive hearing science, processing speed, phonology

## Abstract

**Objective:**

The aim of the present study was to assess the validity of the Ease of Language Understanding (ELU) model through a statistical assessment of the relationships among its main parameters: processing speed, phonology, working memory (WM), and dB Speech Noise Ratio (SNR) for a given Speech Recognition Threshold (SRT) in a sample of hearing aid users from the n200 database.

**Methods:**

Hearing aid users were assessed on several hearing and cognitive tests. Latent Structural Equation Models (SEMs) were applied to investigate the relationship between the main parameters of the ELU model while controlling for age and PTA. Several competing models were assessed.

**Results:**

Analyses indicated that a mediating SEM was the best fit for the data. The results showed that (i) phonology independently predicted speech recognition threshold in both easy and adverse listening conditions and (ii) WM was not predictive of dB SNR for a given SRT in the easier listening conditions (iii) processing speed was predictive of dB SNR for a given SRT mediated *via* WM in the more adverse conditions.

**Conclusion:**

The results were in line with the predictions of the ELU model: (i) phonology contributed to dB SNR for a given SRT in all listening conditions, (ii) WM is only invoked when listening conditions are adverse, (iii) better WM capacity aids the understanding of what has been said in adverse listening conditions, and finally (iv) the results highlight the importance and optimization of processing speed in conditions when listening conditions are adverse and WM is activated.

## 1. Introduction

Cognitive hearing science builds on the principle that individual cognitive functions play an important role from very early post-cochlear and subcortical effects on auditory processing (Stenfelt and Rönnberg, 2009; Sörqvist et al., 2012; Anderson et al., 2013) to interactions among memory systems at cortical levels of listening and understanding speech (Rönnberg et al., 2021). Accepting this tenet, there are important consequences regarding how auditory input processing may be affected. For example, the clinical ramifications of decreased cognitive processing speed may severely impede the compensatory use of higher order cognitive functions such as executive functions and working memory.

Several accounts have been proposed to describe this top-down—bottom-up interaction between auditory input signals and cognitive processing. Among the most studied cognitive concepts is working memory (WM). WM is a temporary memory system measured as an individual’s ability to simultaneously process and temporarily store (dual task) incoming information, also called Working Memory Capacity (WMC; Daneman and Carpenter, 1980; Baddeley, 2012). In the framework of cognitive hearing science, WM represents a hypothetical memory system which provides information required for an individual to process speech in order for communication to occur at the present now. Repeatedly, and especially in adverse listening conditions such as with competing speech maskers (Mattys et al., 2012), individual WMC seems to play a decisive role for speech perception and understanding in general (e.g., Akeroyd, 2008; Rudner et al., 2008). Typically, in this literature the reading span test (RST) has been used as a temporary storage and semantic processing index of WMC (Daneman and Carpenter, 1980). In brief, the participant must listen to a set of short sentences and has for each sentence to make semantic verification judgements, and finally to recall, in the correct sentencewise order, the last (or first) words in the set of sentences.

The strength of the associations between WMC and speech perception and understanding outcomes depends on several factors such as age and hearing impairment (Füllgrabe and Rosen, 2016), auditory temporal fine structure (Bernstein et al., 2016), contextual dependence in the test materials (Rönnberg et al., 2016), and the number of years of hearing aid usage (Ng and Rönnberg, 2020). Furthermore and in relation to conditions that presumably invoke mismatch, WMC has also been studied in relation to syntax processing (Amichetti et al., 2013, 2016; Wingfield et al., 2015), signal processing in hearing aids (Arehart et al., 2013, 2015; Souza et al., 2015), development of phonological/lexical/semantic representations (Luce and Pisoni, 1998; Holmer et al., 2016), its relation to attention (Pichora-Fuller et al., 2016), and priming (Wingfield et al., 2015; Signoret and Rudner, 2019). Moreover, it has been demonstrated that vocabulary is especially important to speech perception in noise (Kennedy-Higgins et al., 2020), either *via* WMC (cf. Janse and Andringa, 2021), for hearing-impaired listeners (Signoret and Rudner, 2019), or in how language is represented in bilinguals (Kilman et al., 2014; Bsharat-maalouf and Karawani, 2022). Needless to say, WM is an important component and predictor variable in the study of cognitive hearing science (Rönnberg et al., 2021), and as we have shown from our research, when the WM test is combined with an inhibition component, then it may be particularly important for Episodic Long-term Memory (ELTM; Sörqvist et al., 2012; Stenbäck et al., 2015).

### 1.1. The ELU model

The Ease of Language Understanding (ELU) model, formulated by Rönnberg (2003) and Rönnberg et al. (2008, 2013) attempts to give a comprehensive account for the role of WM in hearing and builds on previous results/models while attempting to formulate an account of the interaction of different memory systems and early attention mechanisms (Sörqvist et al., 2012). It also accounts for individual variation in the ability to understand and recall speech in adverse listening conditions (Dryden et al., 2017; Mclaughlin et al., 2018). Two overarching processes suggested by the ELU model, both related to WM, determine the communicative competence of the individual: *prediction* of input, and *postdiction*, or reconstruction of what was misheard. Importantly, WM is in this model viewed as interacting with other memory systems to allow postdiction, and for the interaction with an interlocutor (see Rönnberg et al., 2021 for a detailed discussion).

More specifically, the model assumes that explicit WM resources are needed when there is a mismatch between the multimodal phonological representation of the input signal – an episodic buffer (cf. Baddeley, 2000) component named RAMBPHO (Rapid Automatic Multimodal Binding of PHOnology) – and phonological-lexical representations in Semantic Long-Term Memory (SLTM). Mismatch may, e.g., be due to hearing impairment, suboptimal signal processing in the hearing aid or competing speech. The mismatch notion is borrowed from Näätänen et al. (2007), with one important difference. Given a mismatch at lexical access, the ELU model emphasizes the cognitive consequence, *viz.* WM must be involved in postdiction, reconstructing with the help of SLTM, and sometimes Episodic Long-Term Memory (ELTM), what the communicated meaning was by the interlocutor.

In other words, the ELU model builds on the interplay between these three memory processes: WM, SLTM, and ELTM, and a memory resource-the interface RAMBPHO. In easy listening conditions WM acts implicitly to predict input for the listener to understand what has been said through matching input with representations in SLTM. In adverse listening conditions WM acts explicitly to postdict and repair (i.e., infer) what has not implicitly been understood. In the latter condition, WM acts through fragments of temporarily stored RAMBPHO-delivered information and semantic or episodic processing of long-term memory information.

### 1.2. ELU predictions

The ELU model predicts, that in an *easy* implicit listening condition (e.g., where listening is not distracted or obstructed by other adverse sounds physical impairments, and demand on recall), representations of or lexical/semantic meanings are implicitly and automatically unlocked by RAMBPHO input to Long-Term Memory (LTM) and understanding of speech is achieved. Therefore, no effortful top-down processing is necessary. This allows for fast and implicit access to WM in the form of prediction.

However, in *adverse* listening conditions (e.g., where listening is distracted or obstructed by other adverse sounds, physical impairments, or demand on recall), the incoming signal is not optimal by RAMBPHO input and thereby increases the risk that lexical access is not gained directly. To compensate for this mismatch, explicit and deliberate WM processes are invoked, and inference-making based on WM is initiated in interaction with matching syllabic phonological representations in SLTM to reconstruct what lexical entity was implied in the fuzzy input. This process can go back and forth, and therefore takes more time to carry out than automatic and implicit matching from clear input. Examples of WM processes invoked consist of switching of attention, storing of information, inhibiting irrelevant information, semantic integration, and inference-making. In other words, during adverse listening conditions, speech understanding becomes dependent on WMC and is a slower process than when automatic processing occurs, measured in seconds rather than milliseconds.

Mismatch may also occur when lexical access is too slow; for speech understanding to be successful and immediate, processing speed is essential and high demands of lexical processing speed may block lexical access (Rönnberg, 1990, 2003; Ng et al., 2013; Rönnberg et al., 2013). In addition, mismatch may occur when RAMBPHO representations in LTM are not sufficiently precise or stable as a result of long-term severe hearing loss (Andersson and Lyxell, 1998; Andersson, 2002). In individuals with moderate to severe hearing loss, phonological processing has been found to decline, though can be compensated for by WM and phonological WM processes (Classon et al., 2013). In summary, these findings suggest that factors increasing difficulty of hearing such as long-term hearing loss or older age, heightens the individual risk of becoming increasingly dependent on WM in speech processing, thereby strengthening the speech understanding-WM relationship.

Consequently, under mismatch conditions, the prediction from the ELU model is that individuals with high WMC can keep representations in mind and thereby compensate for poor phonological (RAMBPHO) input and/or for poor phonological representations in SLTM, which is also mediated by high lexical access speed. This is maintained in more or less abstracted form to facilitate inferences and intelligent guesswork, cognitive control processes that are not typically ascribed to pure short-term memory (see, e.g., Badre, 2011). In other words, compensation for adverse listening conditions can be accomplished through WM. On the other hand, poorer WMC results in a decreased ability to compensate and consequently a lack of understanding of what has been said/i.e., a higher speech recognition threshold (SRT). See Rönnberg et al. (2013) for an illustration of the ELU model.

It is worth noting that different hypotheses disagree on whether high cognitive capacity results in decreased (Cognitive efficiency hypothesis; ELU model) or increased (Effort hypothesis; Resource hypothesis) processing load (Strand, 2018). Strand (2018) found results supporting the former (in line with the ELU model), where higher cognitive capacity was associated with a decrease in listening effort (listening effort relies on similar cognitive capacities such as WMC). Furthermore, the study assessed whether the impact of cognitive capacity on processing load differed depending on difficult or easy listening conditions but could not find support in line with the ELU model, possibly due to not using a large enough range of Speech Noise Ratio (SNR; +5 or −2 dB; Strand, 2018).

### 1.3. Latent constructs

In the present study, we take a structural equation modelling (SEM) approach to test the original interacting ELU memory system components in explaining the performance of the most traditional clinical outcome variable, the Hagerman matrix test used in Sweden (Hagerman, 1982) used to determine dB SNR for a given SRT through the assessment of the ability to hear different words under different background noise conditions (see 2.2. Procedure). The aim of the present study is therefor to assess the validity of certain parameters of the ELU model and through a hypothesis as well as statistically driven method, construct a SEM model on how they relate to one another. In contrast to other studies, we use the basic components of the ELU model – not just the conditions conducive to explicit WM engagement – but also *processing speed* (the amount of time it takes to perform a cognitive operation; as the operationalization of one property of SLTM), and *phonology* (how sounds blend to form meaning; as the operationalization of RAMBPHO) to predict performance in *dB SNR for a given SRT* (operationalized as Hagerman sentences). Previous studies investigating components of the ELU model have not assessed SLTM, RAMBPHO, and WM in relation to dB SNR for a given SRT in one comprehensive model as a representation of the ELU model, which is the aim of the present study.

However, a recent study (Janse and Andringa, 2021), also using a SEM approach, investigated whether WM, processing speed, vocabulary knowledge and hearing acuity independently accounted for the variance of word identification in fast speech among older individuals. Their findings showed that only WM and hearing acuity were associated with word recognition (Janse and Andringa, 2021), and the relationship between WM and word recognition was at odds with the present study in that the association was weaker rather than stronger in the more adverse listening conditions. The authors align their finding with theories suggesting that WM contains activated LTM information and that there is no structural difference between WM and LTM (MacDonald and Christiansen, 2002; Cowan, 2005); suggesting that high WM is a result of being able to process language efficiently, not vice versa. Therefore, more adverse listening conditions would hinder the activation of WM. It is of interest to compare their findings to the present study as their results are not in line with the prediction of the ELU model. In addition, the present study uses a sample of hearing aid users while their sample consisted of non-users of hearing aids and while both studies assess WM and processing speed and a similar outcome, vocabulary knowledge was assessed in Janse and Ardringas study while in the present phonology was assessed. Because of these differences, in a SEM approach, interesting but diverging results may be expected.

#### 1.3.1. The ELU SEM

The relationships of the parameters were based on the predictions of the ELU model. Firstly, we assumed a model where RAMBPHO/phonology independently predicted individual dB SNR for a given SRT. This assumption was based on that if incoming information was abstracted in RAMBPHO and matched with representations in SLTM, perception and understanding would occur with ease. Secondly, we hypothesized that speed of processing as the operationalization of one property of SLTM, may be predictive of dB SNR for a given SRT under certain listening conditions (adverse listening conditions leading to mismatch) as speed of processing is essential for lexical access. Finally, we hypothesized that WM was predictive of individual dB SNR for a given SRT. But we also assumed that, in line with the ELU model, processing speed was predictive of WM as speed of processing is essential for lexical access. Speed of processing is therefore also of use in the WM-SLTM interactions under mismatch conditions as an increase in interactions between WM-SLTM is necessary in order for postdiction to result in a correct prediction. Additionally, processing speed is then also of importance as to move between alternatives in the mismatch process in WM. Or in other words, a mediation model was assumed where speed of processing was mediated *via* WM to predict the dB SNR for a given SRT outcome.

In line with the literature, the present study assumed that the conditions that put a pressure on lexical access speed are when speech maskers compete with the target materials (due to informational masking, especially for four talkers (4T, i.e., being able to pick up lexical information rapidly in small time-windows when the masker speech is not present) between peaks of amplitude modulations of speech (Lunner et al., 2009; Ng and Rönnberg, 2020) and when recall demands are high in the matrix test by Hagerman (80 vs. 50% to be recalled). Without such pressure, lexical access speed is less critical. This is also crucially reflected in the correlation with WMC, which only then predicts Hagerman sentences performance.

Moreover, the Hagerman test allows us to assess an important aspect of the ELU model, namely whether and how WM is used in easy and adverse listening conditions. The Hagerman test consists of several conditions which vary in difficulty through the use of easy and more adverse background noise and through the use of different thresholds. The presently postulated SEM model allows for the assessment of whether and how the engagement of the present parameters vary in easy and adverse listening conditions.

The objective of the present study is therefore to test whether the predictions of the ELU model can be explained through a mediation SEM model testing its parameters. A SEM model is suitable as it allows the investigation of multiple relationships simultaneously, presently this means the investigation of which cognitive capacity/capacities independently accounts for dB SNR and weather and how these capacities are interrelated. The assessment of the current model is also based on the ELU assumption of individual differences in cognitive hearing, and the presently assessed structures have all been shown to be associated with dB SNR separately: WM (Akeroyd, 2008), processing speed (Dryden et al., 2017), and phonology (Lyxell et al., 1998).

The present sample used to test the predictions of the ELU model (n200), was specifically designed to assess the validity of the ELU model (Rönnberg et al., 2016). Whether WM is associated with the ability to understand speech has been examined in this sample previously and has been supported (Ng and Rönnberg, 2020). However, the current study develops this by including RAMBPHO and processing speed to assess how well these components support the above outlined predictions of the ELU model.

### 1.4. Alternative model predictions

As competing or complementary predictions can be derived from other cognitive accounts alternative models were tested as to assess whether other alternative relationships between the parameters were a more viable option than what is presently assessed and proposed by the ELU model. While both the Baddeley and Daneman and Carpenter models have influenced the successive build-up of the ELU model, we here focus on some alternative predictions with an anchor in the cognitive aging literature, based on the same variables used to generate the constructs used in the modelling of the ELU predictions. Thus, by re-grouping the test variables used, into new, alternative prediction combinations, we are in a position to make some comparisons. We are however aware that there are other alternative models (e.g., Trace, NAM), but the parameters of the present study were not sufficient to assess these models and therefore it was deemed that assessment of these models would be unjustified.

Since our sample is of a relatively older age (mean age 61.57 years), we can test a few alternative notions derived from the cognitive aging literature. One obvious candidate is to test a general speed account (Salthouse, 1996). This can easily be done by pooling both the latent phonology and processing speed concepts, since all four indices are latency measures (see under method), and they would approximate some of Salthouse’s claim about a general speed parameter. Thus, in this alternative model 1, General Speed (GS) is set to predict Hagerman sentences overall. A modified version (alternative model 2) would be to combine GS with WM (which is a combination of RST, and one visuo-spatial, and one word-pair test dual task test, see method). The models are run with age and hearing loss partialled out but also included as they are intimately tied to aging, and cognitive aging.

A general model builds on Baddeley’s notion of adding an episodic buffer to WM (Baddeley, 2000). With the current set of variables, we could construct a General WM model (GWM) by adding RAMBPHO (phonology) to the WM dual tasks (alternative model 3). Speed could then be added to investigate all variables in the same test run (alternative model 4).

## 2. Methods

### 2.1. Participants

We used data from the n200 study of 200 hearing impaired hearing aid users (Rönnberg et al., 2016). Participants were acclimatized hearing aid users recruited randomly from Linköping University Hospital in Sweden. Participants had bilateral, symmetrical mild to severe sensorineural hearing loss. For a detailed description of the n200 study see Rönnberg et al. (2016). All participants gave their informed consent. The study was given ethical approval by its regional committee.

### 2.2. Procedure

Several cognitive and hearing tests were conducted in the n200 study by clinical audiologists (Rönnberg et al., 2016). Participants were assessed while hearing aids were being worn. The present study included a subset of these including tests on dB SNR for a given SRT, WM, phonology, and processing speed. These structures (processing speed, phonology, WM) were constructed in line with a factor analysis previously performed on the n200 data (Rönnberg et al., 2016) as to investigate possible latent factors. The tests included in the present study were all included in the previous factor analysis where they all significantly loaded onto its respective factor, providing support for the structure of tests in the present study.

***Working memory*** consisted of 3 tests in the present study including verbal and non-verbal tasks-the RST, Semantic word pair span (SWPST), and Visuo-spatial WM test (VSMW; Rönnberg et al., 1989; Lunner, 2003; Foo et al., 2007). All WM assessments are dual task assessments, meaning that they assess both WM processing and storage at the same time and assume that the higher the demand on WM for processing, the less WM is available for storage. Dual tasks are utilized as previous findings have shown that it is the dual nature of the WM task that is an important aspect, and not just the serial recall aspect. The storage AND processing aspect have been proven to be more crucial to speech in noise performance (Rönnberg et al., 2016).

In the *RST*, participants were presented with three short word sentences on a computer screen, one word at a time, and asked to judge whether the sentence made sense or not. Sentences were presented with increasing difficulty in sets of two to five. After each set of sentences, the participant was asked to recall the first or last word in the skriv ihop sentence wise presentation order. The test was scored based on the number of total recalled words irrespective of order. The maximum score was 28.

In the *VSMW* participants were assessed on their ability to recall non-verbal WM, information. Initially participants were presented with a 5×5 grid of squares on a screen. In one square of the grid, identical or different shapes were presented, and participants were asked to judge whether ellipses were identical or not. After a response the same task was repeated in a different square of the grid. This was continued until the end of the list which varied from two to five pairs and three trials per length. The total amount of administered trials was 42. After each list was completed, participants were asked to draw on a replicated 5×5 grid, the location, and the correct order of presentation of the shapes as they recalled them. The test was scored based on the total number of squares recalled and the maximum score was 42.

In the *SWPST*, participants were presented with pairs of words on a screen and asked which of the two words represented a living object. After one set of word pairs, the participants were asked to recall the first or second of the words. The test evaluates WM capacity which does not involve syntactic elements in the processing and storage components. The test was scored based on the number of total recalled words irrespective of order with a maximum score of 42.

***Phonology*** consisted of two tests in the present study, rhyme (speed), and Gating. RAMBPHO is necessarily a broad concept since it encompasses the integration of several modalities which deliver phonetic and phonological information in different ways and with different relative timing. This takes into consideration natural conversations and not only modality specific phonological information. The motivation behind using gating tasks and rhyme speed (in the latent construct phonology) is to capture some more abstract and integrated phonological representations, early and a little later in the RAMBPHO abstraction process. In *the rhyme test*, the participants were presented with two words and asked to determine whether they rhymed or not regardless of spelling (example: hat-bat, find-shoe). Accuracy and response time was measured in *ms*, and response time was used in the present study. The *gating* paradigm (Grosjean, 1980; Moradi et al., 2013, 2014a,b, 2016) assess early identification of phonetic information. In the task, participants were asked to identify the vowel in a consonant-vowel-consonant syllabus combination and the consonant in a vowel-consonant-vowel syllabus combination. The test measures the duration of the signal required for speech recognition in *ms*. In the present study, isolation points (IPs) were used as an outcome, that is the proportion of a signal required for its correct identification (vowel and consonant).

***Processing speed*** consisted of the amount of time (rt) it took to complete the following two tests in the present study: lexical decision and physical matching. We used physical matching and lexical decision speed to capture speed in dealing with verbal and lexical retrieval aspects of SLTM. Many other aspects of SLTM do exist but the data-base focused on tests that would capture the original formulation of components of the ELU model. In *the Physical Matching task*, the participants were presented with two letters on a screen and asked to determine whether the letters were identical or not (for example: A-a, A-A). In *the Lexical Decision task*, the participants were presented with a three-letter combination and asked to determine whether it was a real word or a nonsense word (for example: she, vni). All words in all the tests were in the participants’ native language (Swedish) and the real words were all familiar Swedish words. In all the tests the participant responded by pressing a yes or a no button and accuracy and response time in *ms* was recorded.

***dB SNR for a given SRT*** was measured using Hagerman sentences (Hagerman, 1982; Hagerman and Kinnefors, 1995). In the Hagerman test, the participants were asked to identify specific words in different noise conditions to obtain a dB SNR for a given SRT. These matrix sentences consist of lists of 10 five-word low redundancy sentences, all heard with a background noise, using commonly used Swedish words (Allén, 1970). It is important to note that the words cannot be predicted or guessed by the participant but needs to be heard clearly to be understood. There were two main background noise conditions: Four Talker babble (4T) and Speech Shaped Noise (SSN). In the 4T condition, the background noise consists of 4 people (2 men and 2 women) talking at the same time (reading aloud from a newspaper). In the SSN condition, the background noise was an amplitude modulated speech weighed noise. 4T is considered to be a more adverse listening condition than SSN (Kilman et al., 2014). After each sentence participants were asked to identify the five words in each sentence and verbally repeat them.

The sound level was initially set at 65 dB SPL targeting SRT. The procedure was adaptive: the SNRs was increased or decreased by 1 dB after each task depending on the performance of the participant in identifying the words. If the participant could identify the words in each task, the background noise level was increased in the following task, and thereby also the difficulty in identifying the words. If the participant could not identify the words, the noise level in the following task was decreased, thereby making the task easier. Specifically, if the word recognition in a sentence was 2 words, there was no change in signal to noise ratio (dB SNR). If word recognition was below two (zero or one identified words), the noise level in the following sentence was decreased (by 2 and 1 dB, respectively). If instead 3, 4, or 5 words were recognized, the noise level was increased by 1, 2, or 3 dB, respectively.

In addition to two different types of background noise, the Hagerman matrix test also applies target SRTs of 50- or 80%-word recognition, where 50/80% is the threshold required in recognized words for the noise level to be increased. The 50 and 80% conditions were alternated for each list with an equal number in each of the condition. A higher threshold in the 80% condition where 80% of the words need to be recognized in order to increase the background noise. Note that the above description of required recognized words as to change noise background levels apply to the 50% condition, in the 80% condition 4 correctly recognized words are required as to increase background noise level.

Participants were given practice rounds of 2 lists of 10 sentences each as this reduces any training effect (Hagerman and Kinnefors, 1995). Performance was calculated based on average dB SNR across sentences. Moreover, three signal processing conditions were used: linear amplification with and without noise reduction, and fast compression with no noise reduction. Three lists per signal processing condition was used. However, the focus of the present study was not on signal processing and the different conditions were therefore not separated out. The outcome variable in the present study was therefore dB SNR for a given SRT-the individual strength of the signal to noise ratio required to reach the 50% (correctly recognized words) or 80% (80% correctly recognized words) in 4T or SSN background noise. The method used to present the Hagerman sentences was an interleaved method (Brand, 2000) where 50 and 80% SRT was the goal of every second sentence – they were alternated in the same list. An equal number of sentences were used to reach 50 and 80% threshold, respectively.

### 2.3. Statistical analyses

Latent Structural Equation Models (SEM) were applied to investigate our predictions and to avoid shortcomings of testing singular aspects of relationships between variables. SEM models measure structural relationships between variables and encompasses a combination of factor analyses, correlations, and multiple regression analysis. In the present study, latent variables were constructed within a SEM model where relationships were allowed between latent constructs (measures loading onto each latent construct are written in brackets) of *processing speed* (lexical matching, physical matching), *phonology* (rhyme, gating), *WM* (RST, SWPST, VSMW), and our outcome measure of dB SNR for a given *SRT* (different combination of the Hagerman tests). No parceling was necessary as each latent construct was defined by more than one indicator. In line with the advantages of SEM, our aim was to assess whether out latent constructs (WM, processing speed, and phonology) were indicators of dB SNR for a given SRT as well as whether our latent constructs predicted individual differences in dB SNR for a given SRT; SEM assesses both structural and measurements models in combination.

Initially, hypothesized competing models (see 2.2. Procedure) were assessed as to find the best fitting model to the data, in line with general recommendations of data fitting procedures (see Alternative models; Goodboy and Kline, 2017). A multitude of models were assessed and only a selection is reported here. The ones reported are both hypotheses driven as well as a better fit of the data compared to other models. Models assessed were (amongst others) (i) A model where all tasks based on speed where combined into one latent speed factor [called General speed (GS); including phonology and speed tasks] and allowed to predict the outcome Hagerman sentences [Alternative Model 1 (AM1); Salthouse, 1996], (ii) A model where GS and WM were allowed to independently predict Hagerman sentences (AM2), (iii) A model where WM and phonology were combined into one latent construct (GWM) in line with Baddeley’s (Baddeley, 2000) inclusive WM concept where RAMBPHO acts as an episodic buffer (AM3), (iv) A model allowing GWM and speed to independently predict the outcome (Hagerman sentences; AM4). In addition, the VSWM task in the latent WM construct was included and excluded in all the above models as to assess whether solely assessing verbal tasks, in line with outcome measure which only assess verbal tasks, had an effect. In addition, a model where all constructs were allowed to independently predict the outcome was assessed (AM5). In line with our final mediation model, all models were run with and without covariates (Age and PTA).

Secondly, we defined and assessed the main model of the study based on our hypothesis of the relationships between processing speed, WM, phonology, and Hagerman sentences (see 2.2.Procedure). Several SEM models were run where processing speed and phonology were allowed to independently predict the outcome (Hagerman sentences). Processing speed was predicted to be mediated *via* WM to Hagerman sentences, mediation models were therefore performed. SEM models are appropriate when the interest lies on the relationships between different factors. Mediation models are of interest in understanding underlying mechanisms as it clarifies how particular factors impact an outcome. An alternative approach are regression models, however these are ill-suited as they assume variables as either cause or effect, while the underlying assumption in the present study are in line with SEM models in that all variables may be both causes and effects. Benefits of SEM models are (i) the assessment of fit of data to a hypothesized model, (ii) estimation of data to latent variables, and (iii) assessment of measurement error. For an in-depth description of mediation models (see Baron and Kenny, 1986; Muthén and Asparouhov, 2015; Lee et al., 2021).

Initially our main model assessed a mediation model including all the Hagerman test conditions (4T, SSN, 50 and 80% thresholds; Model 1). Moreover, in line with the ELU model, we hypothesized that WM would only be called upon in adverse listening conditions. As the Hagerman test vary in difficulty and noise level, or in other words, how adverse the listening conditions are, the study provides the opportunity to assess whether the involvement of WM differs depending on different conditions. The 4T condition is considered a more adverse listening condition compared to the SSN and the 80% threshold presents a more difficult assessment compared to the 50% threshold (see Rönnberg et al., 2016 for a detailed description of the variation of dB SNR for a given SRT based on level of difficulty). To treat 4T and SSN separately is generally supported as a stronger relationship exists between WM and 4T conditions than between WM and the SSN condition (Ng and Rönnberg, 2020). However, while 80% SRT is generally considered to be the more difficult, condition compared to 50% SRT, and shown to be associated with WM where the 50% SRT is not (Lunner and Sundewall-Thorén, 2007; Larsby et al., 2008), it has also been shown that WM can play a role in the 50% SRT condition in adults (Gordon-Salant and Cole, 2016).

**Figure 1:**
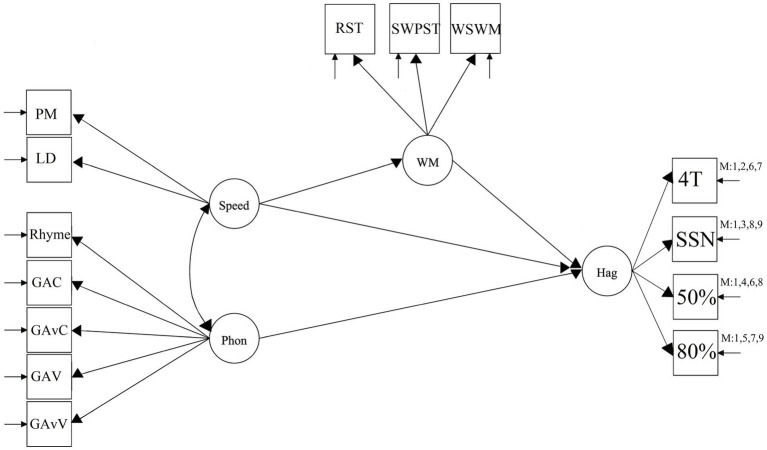
SEM mediation model where speed is mediated *via* WM and Speed and Phon (phonology) allowed to directly predict outcome-Hag (Hagerman). Phonology and speed are allowed to correlate. Speed is predicted by PM (Physical matching speed) and LD (Lexical Decision speed). Phonology is predicted by Rhyme and Gating conditions (G = Gating, AC = Audio Consonant, AvC = AudioVisual Consonant, AV = Audio Vocal, AvV = AudioVisual Vocal, i.e., a RAMBPHO composite). WM is predicted by RST (Reading Span Task), SWPST (Semantic Word Pair Span), and WSWM (Visuo-spatial WM test). Hag = Hagerman and is predicted by all the different conditions of the Hagerman test including 4T (Four Talker Babble), SSN (Speech Shaped Noise), 50/80% threshold. M: followed by numbers represents the different conditions included in the different models. The disconnected arrows indicate that the model accounts for residuals.

It is therefore of interest to systematically assess the different conditions as it is unclear which conditions and in which combination they may be involved with WM. We, therefore, ran different models including a variation of the different Hagerman conditions to assess whether the conditions of 4T and SSN as well as 50 and 80% significantly differed in how the parameters related to one another and whether they did so in different combinations. The following Hagerman test outcomes were included in separate models: (1) All Hagerman test items, (2) 4T only, (3) SSN only, (4) 50% only, (5) 80% only, (6) 4T at 50% only, (7) 4T at 80% only, (8) SSN at 50% only, and (9) SSN at 80% only. In model 6–9, 4T and SSN was divided between 50 and 80% as we expected that 4T and SSN would indicate significant differences due to a difference in the level of difficulty. To therefore combine both 4T and SSN in the 50 and 80% conditions may not show any clear results and the different conditions were therefore systematically assessed in separate models as to assess whether patterns across the different conditions could be observed. The estimated base model, without regression weights (as the aim is not to find a best fitting model but to compare different Hagerman conditions), is presented in Figure 1.

Models were run with and without covariates where age and four frequency pure tone average (PTA; for the better ear.5, 1, 2, and 4 kHz) were used as covariates. Controlling for age and hearing loss results in a more general model of the parameters underlying the ELU model, which is the main purpose of the paper. If age and hearing loss is not controlled for, separate models for the different groups may be required, which has shown to not be crucial (Marsja et al., 2022). This resulted in 18 different models, labelled M1-M9 where models including covariates were labelled with the model number followed by a (see Table 1 for the different models and their results). An initial assessment of whether 4T and SSN as well as 50 and 80% differed was made using a t-test.

**Table 1:** Model fit indices of competing alternative models.

Model	χ^2^	df	χ^2^/df	RMSEA	CFI	TLI	SRMR	R2	BIC	AIC
AM1. GS → Hag	959.37	212	4.52	0.145	0.622	0.588	0.295	0.10*	15023.61	14826.80
AM1a.	981.15	252	3.89	0.132	0.643	0.610	0.224	0.40***	14897.14	14688.24
AM2. GS + WM → Hag	862.94	207	4.17	0.137	0.668	0.629	0.278	0.20*	14952.80	14740.37
AM2a.	897.34	246	3.65	0.126	0.681	0.643	0.210	0.45***	14844.05	14616.43
AM2^1^.	838.57	187	4.48	0.144	0.661	0.654	0.291	0.19*	13966.54	13763.39
AM2^1^a.	863.24	224	3.85	0.131	0.679	0.623	0.219	0.43***	13846.48	13628.22
AM3. GWM → Hag	424.95	169	2.51	0.095	0.855	0.837	0.098	0.68***	13788.64	13598.08
AM3a.	463.17	205	2.26	0.087	0.856	0.839	0.095	0.66***	13715.98	13513.31
AM3^1^.	381.97	151	2.53	0.095	0.866	0.848	0.084	0.67***	12766.35	12585.16
AM3^1^a.	417.96	185	2.26	0.087	0.868	0.851	0.085	0.65***	12693.28	12499.97
AM4. Speed + GWM → Hag	605.37	207	2.92	0.107	0.798	0.760	0.117	0.66***	14695.23	14482.80
AM4a.	664.35	246	2.70	0.101	0.795	0.756	0.119	0.66***	14611.06	14383.45
AM4^1^.	546.09	187	2.92	0.107	0.813	0.799	0.106	0.64***	13674.07	13471.01
AM4^1^a.	604.91	224	2.70	0.101	0.809	0.787	0.112	0.65***	13588.15	13369.89
AM5. Speed + WM + Phon → Hag	547.02	204	2.68	0.100	0.826	0.803	0.106	0.29***	14652.25	14430.49
AM5a.	592.43	240	2.47	0.094	0.827	0.802	0.099	0.31***	14569.84	14323.52
M1	366.87***	199	1.84	0.071	0.915	0.901	0.110	0.60***	14,497–72	14260.30
M1a	417.15***	235	1.77	0.068	0.911	0.896	0.104	0.69***	14420.15	14158.24
Models of subsets of Hagerman conditions										
M2	216.53***	98	2.20	0.085	0.896	0.873	0.084	0.72***	11006.48	10837.78
M2a	248.88***	122	2.04	0.079	0.895	0.869	0.109	0.73***	10923.31	10729.99
M3	205.37***	97	2.11	0.082	0.912	0.891	0.123	0.72***	10904.06	10732.24
M3a	242.10***	121	2.00	0.077	0.906	0.882	0.112	0.69***	10836.56	10640.12
M4	204.66***	98	2.09	0.080	0.922	0.905	0.127	0.74***	10213.29	10044.60
M4a	242.69***	122	1.98	0.077	0.916	0.895	0.114	0.72***	10143.03	9949.72
M5	199.46***	98	2.04	0.079	0.901	0.882	0.109	0.60***	11622.42	11453.72
M5a	235.95***	122	1.93	0.075	0.898	0.873	0.099	0.62***	11544.75	11351.43
M6	147.70***	59	2.50	0.095	0.901	0.869	0.125	0.75***	8854.19	8713.62
M6a	176.03***	77	2.29	0.088	0.896	0.860	0.113	0.74***	8783.28	8618.02
M7	145.04***	59	2.45	0.093	0.891	0.867	0.115	0.63***	9422.53	9281.95
M7a	174.30***	77	2.26	0.087	0.886	0.856	0.104	0.69***	9344.08	9178.83
M8	155.95***	59	2.64	0.099	0.900	0.868	0.135	0.74***	8706.11	8565.53
M8a	183.47***	77	2.38	0.091	0.895	0.859	0.121	0.71***	8642.45	8477.20
M9	142.78***	59	2.42	0.092	0.893	0.859	0.122	0.59***	9654.34	9532.23
M9a	172.04***	77	2.23	0.086	0.886	0.847	0.110	0.59***	9440.32	9275.07

AM = alternative model; GS = general speed; Hag = Hagerman; GWM = phonology + WM; a = covariates (PTA and Age);

1 = excluding VSWM. All models represent a latent construct as a combination of the different latent constructs of interest, allowed to predict the outcome measure (Hagerman).

* *p* < 0.05;

** *p* < 0.01;

*** *p* < 0.001.

STATA 14 was used for data preparation and SEM mediation models were conducted using Mplus 8 (Muthén and Muthén, 2013). Modification indices were adjusted for if significant in each model. SEM models test whether the hypothesized model is a good explanation of the data, and several fit estimators are used to assess this. SEM models are typically evaluated against several fit indices, that assess different aspects. The fit indices reported differ between articles and between guidelines. However, we had picked a set of fit indices (RMSEA, CFI, TLI, and SRMR) that cover the most relevant aspects (see Schreiber et al., 2006). RMSEA assess absolute fit index, CFI incremental fit index and SRMR assess exact fit. A good fit is indicated by RMSEA value of <0.06, SRMR of <0.05, and CFI of >0.95 while a satisfactory fit is indicated by an RMSEA value of <0.08, SRMR <0.08, and CFI >0.90 (Jöreskog and Sörbom, 1993; Hu and Bentler, 1999; Hooper et al., 2008; Awang, 2012). TLI values of >0.90 or >0.95 indicate an acceptable fit (Bentler and Bonett, 1980; Hu and Bentler, 1999). Chi-square values are also reported. However, chi-square is not always reliable depending on sample size (MacCallum et al., 1996) and therefore the normed or relative chi-square was reported (chi-square/df) which is less sensitive to sample size. The criterion for acceptance varies but is at it strictest lower than 2 (Ullman, 2001) for an acceptable model fit while a more liberal estimate is lower than 5 (Schumacker and Lomax, 2004). BIC and AIC values were also reported and used for model comparisons where the model with the lowest values are preferred (Hastie et al., 2016). It should be noted that AIC was not designed to compare non-nested model (Akaike, 1973), however, both BIC and AIC values are presently reported for model comparison as BIC tends to favor less complex models while AIC tends to favor more complex models (Murphy, 2012). Alternative models were compared to our main hypothesized model based on the following model fit estimators. It should be noted that our goal with model fit estimators regarding our main model assessing the ELU model was not to find a best fitting model. The below fit indices were therefore here used to understand whether the model represented the data or not. ML estimator was used. Speed variables measuring rt. were transformed into z-scores in the SEM model.

## 3. Results

### 3.1. Descriptive

Participants were removed if they had less than 2 years of hearing aid experience (*n* = 22), based on that it takes time to acclimatize to their hearing aids processed signals and therefore individuals with less than 2 years of hearing aid use may not be comparable (Ng et al., 2014; Ng and Rönnberg, 2020). Participants were also removed if they had no reported data on length of hearing aid use (*n* = 17). In the Hagerman matrix test, results were considered unreliable, and individuals were excluded if the individual curve between the 50 and 80% levels of performance indicated a slope of 2% or below (Foo et al., 2007; Ng and Rönnberg, 2020; *n* = 4). Participants were also excluded if there were outlier points of 4 SD above or below the mean (*n* = 4). This resulted in a total sample of 168 hearing aid users (*n* = 91 male). Missing data points were minimal to none in all the assessments and background information.

The mean age of the participants was 61.57 years (SD = 8.11, range 35–80 years). Average time of having hearing problems was 14.29 years (11.09, range 2–65 years) while the average time of having a hearing aid was 7.40 years (SD = 6.78, range 2–45 years). Mean age of education was 13.29 years (SD = 3.59, range 6–25.5). Less than half the sample were female (*n* = 77). About half of the included sample were still employed (*n* = 86), and half retired (*n* = 79; *n* = 2 unemployed and *n* = 1 student). The majority of the participants were cohabiting or were married (*n* = 143). About two-thirds of the sample had tinnitus (*n* = 99). All participants were native Swedish speakers, and all had normal or corrected-to-normal vision.

Descriptive statistics of measurements used in the SEM model are presented in Table 2. A manipulation check with t-tests of the Hagerman test conditions confirmed that the intended more adverse conditions were harder than the intended easier conditions [4T50 vs. 4T80, *t*(155) = 39.34; *p* < 0.001; SSN50 vs. SSN80, *t*(155) = 36.80, *p* < 0.001; 4T50 vs. SSN50, *t*(155) = 73.98, *p* < 0.001; and 4T80 vs. SSN80, *t*(155) = 35,87, *p* < 0.001]. Correlations between tasks in the same latent variable were mostly medium to large, which makes a SEM appropriate, see Table 3.

**Table 2:** Descriptive statistics of measures used in the analysis (*n* = 168).

	Mean	SD	Range
Hagerman^1^			
Hagerman total	−2.92	1.77	−6.15; 1.69
Hagerman Fast 4T50	−0.25	1.97	−6.78; 6.11
Hagerman Fast 4T80	4.97	2.93	−0.44; 14
Hagerman Fast SSN50	−5.95	1.63	−10; −1.67
Hagerman Fast SSN80	−0.54	3.49	−7.11; 11.67
Hagerman NR 4T50	−7.96	1.54	−11.22; −2.22
Hagerman NR 4T80	−2.50	2.80	−7.56; 6.44
Hagerman NR SSN50	−11.32	1.52	−15.11; −5.33
Hagerman NR SSN80	−5.82	3.08	−10.67; 4.22
Hagerman NP 4T50	−0.96	1.77	−8.11; 3.22
Hagerman NP 4T80	3.70	2.86	−1.78; 11.11
Hagerman NP SSN50	6.27	1.53	−10.78; −1.89
Hagerman NP SSN80	−2.02	2.80	−7.33; 6.56
Working memory			
Semantic word pair	17.3	5.22	3;33
Reading span	16.06	3.70	5;26
VWMW span	29.18	6.10	9;42
Phonology			
Rhyme rt. (ms)	1717.13	411.95	1,004; 3,204
Gating^2^ A C	7.72	3.32	2.4; 16
Gating Av C	5.58	2.95	2.8; 16
Gating A V	5.85	2.50	1.2; 11.4
Gating Av V	5.55	2.44	1.8; 11.4
Speed			
Lexical decision rt. (ms)	987.50	209.80	674; 1906
Physical matching rt. (ms)	989.41	206.94	552; 1,575
Covariates			
PTA better ear	38.26	11.08	5; 75
Age	61.57	8.11	35; 80

1 Fast = fast compression no noise reduction, NR = noise reduction linear amplification, NP = No processing-linear amplification with no noise reduction.

2 Gating: A = Audio, Av = audio visual, C = Consonant, V = vocal (measured in isolation points).

**Table 3:** Cross-task correlations across tasks for each latent construct.

Latent construct		WM			Speed		Phonology				
	Task	VSWM	RST	SWPST	LD	PM	Rhyme	G AC	G AvC	G AV	G AvV
WM	VSMW	-									
	RST	0.37***	-								
	SWPST	0.35***	0.40***	-							
Speed	LD	−0.16*	−0.28**	−0.28**	-						
	PM	−0.39***	−0.26**	−0.22*	0.54***	-					
Phonology	Rhyme	−0.13	−0.29***	−0.35***	0.71***	0.38***	-				
	G AC	−0.16*	−0.18*	−0.18*	0.31***	0.31***	0.26**	-			
	G AvC	0.05	−0.11	−0.16*	0.28***	0.12	0.26**	0.71***	-		
	G AV	−0.15	−0.19*	−0.20**	0.26***	0.17*	0.23**	0.56***	0.56***	-	
	G AvV	−0.16*	−0.25**	−0.27**	0.33***	0.26**	0.34***	0.51***	0.62***	0.71***	-
Hagerman	FA4T50	−0.18*	−0.13	−0.21**	0.24**	0.30**	0.13	0.21*	0.39***	0.24**	0.19*
	FA4T80	−0.30	−0.20*	−0.11	0.06	0.15	0.12	0.20*	0.20*	0.15	0.09
	FASSN50	−0.27**	−0.22**	−0.18*	0.34***	0.38***	0.24**	0.34***	0.42***	0.37***	0.29**
	FASSN80	−0.16*	−0.07	−0.08	0.28**	0.19*	0.24**	0.24**	0.31***	0.14	0.16
	NP4T50	−0.24**	−0.17*	−0.15	0.21**	0.26**	0.13	0.36***	0.24**	0.41***	0.28**
	NP4T80	−0.27**	−0.20*	−0.28**	0.19*	0.19*	0.16*	0.35***	0.21*	0.31***	0.25*
	NPSSN50	−0.18*	−0.16*	−0.17*	0.27**	0.25**	0.19*	0.44***	0.39***	0.40***	0.24*
	NPSSN80	−0.19*	−0.17*	−0.12	0.15	0.15	0.10	0.29**	0.19*	0.31***	0.19*
	NR4T50	−0.27**	−0.28**	−0.25**	0.22**	0.29**	0.15	0.40***	0.38***	0.38***	0.29**
	NR4T80	−0.19*	−0.08	−0.19*	0.14	0.11	0.06	0.32***	0.14	0.28**	0.15
	NRSSN50	−0.21**	−0.21**	−0.16*	0.25**	0.24**	0.15	0.27**	0.21*	0.36***	0.30***
	NRSSN80	−0.25**	−0.25**	−0.17*	0.29**	0.25**	0.26**	0.28**	0.18*	0.27**	0.29**

Latent construct		Hagerman											
	Task	FA4T50	FA4T80	FASSN50	FASSN80	NP4T50	NP4T80	NPSSN50	NPSSN80	NR4T50	NR4T80	NRSSN50	NRSSN80
WM	VSMW												
	RST												
	SWPST												
Speed	LD												
	PM												
Phonology	Rhyme												
	G AC												
	G AvC												
	G AV												
	G AvV												
Hagerman	FA4T50	-											
	FA4T80	0.52***	-										
	FASSN50	0.70***	0.53***	-									
	FASSN80	0.60***	0.54***	0.63***	-								
	NP4T50	0.57***	0.46***	0.68***	0.39***	-							
	NP4T80	0.54***	0.53***	0.50***	0.55***	0.62***	-						
	NPSSN50	0.61***	0.47***	0.74***	0.59***	0.72***	0.65***	-					
	NPSSN80	0.47***	0.49***	0.56***	0.54***	0.51***	0.54***	0.54***	-				
	NR4T50	0.64***	0.47***	0.69***	0.44***	0.67***	0.60***	0.71***	0.47***	-			
	NR4T80	0.42***	0.46***	0.38***	0.41***	0.39***	0.45***	0.46***	0.45***	0.51***	-		
	NRSSN50	0.55***	0.42***	0.67***	0.51***	0.67***	0.56***	0.71***	0.44***	0.66***	0.44***	-	
	NRSSN80	0.40***	0.45***	0.55***	0.48***	0.51***	0.60***	0.56***	0.44***	0.60***	0.51***	0.66***	-

RST = reading span task, SWPST = semantic word pair span, WSMW=visuo-spatial WM test, PM = physical matching speed. LD = lexical decision speed, G = gating, AC = Audio consonant, AvC = Audiovisual Consonant, AV = audio vowel, AvV = Audiovisual Vowel. Hagerman: FA = fast compression with no noise reduction, NP = linear amplification without noise reduction, NR = linear amplification with noise reduction, 4T = four Talker babble, SSN=Speech Shaped Noise, 50/80 = SRT.

* = *p* < 0.05;

** = *p* < 0.01;

*** = *p* < 0.001

### 3.2. SEM results

Fit indices for all SEM models (with and without covariates) are presented in Table 1. The interpretation of models will focus on the models with covariates, which typically had a better fit than the models without covariates. The ELU model had acceptable fit on most of the fit indices, but SRMR was just outside the cut-off. None of the alternative models reached an acceptable fit of the data according to RMSEA, CFI, TLI or χ^2^/df. AM1 indicated the worse fitting model. Adding speed to this model (AM2) improved the model slightly but still indicated a very poor explanation of the data. According to RMSEA, CFI, TLI, χ^2^/df, BIC, and AIC, the alternative model which best explained the data was model 3a-a model combining phonology and WM while excluding VSWM (Baddeley’s General WM model). Adding speed to this model decreased the model fit. Finally, AM5 indicated a poor fit of the data according to all fit indices.

A model representing the ELU model (M1) where speed and phonology were allowed to independently predict the outcome and speed was mediated *via* WM to the outcome, indicated an acceptable fit and a better fit of the data compared to all the alternative models. In this model all the Hagerman test conditions were included. The results indicated a model where phonology predicted Hagerman sentences and where speed was mediated *via* WM to Hagerman sentences. Phonology and speed were significantly correlated. Speed did not independently predict Hagerman sentences. The results of the relationships between variables [correlations and regressions (paths)] are presented in Table 4.

**Table 4:** Results of mediation analyses.

Model	Outcome	Group	Significant predictors of outcome				
			Phon → Hag	Speed → Hag	Speed → WM	WM → Hag	Phon-Speed
M1	All	No covariates	0.79***	−0.01	−0.49***	−0.22**	0.25**
M1a		Covariates	0.62***	−0.05	−0.39***	−0.19*	0.23**
M2	4T	No covariates	0.78***	−0.05	−0.49***	−0.29***	0.25**
M2a		Covariates	0.59***	−0.10	−0.39***	−0.26**	0.24**
M3	SSN	No covariates	0.80***	0.02	−0.49***	−0.18*	0.25**
M3a		Covariates	0.66***	−0.03	−0.39***	−0.14	0.23**
M4	50	No covariates	0.81***	0.02	−0.49***	−0.19*	0.25**
M4a		Covariates	0.66***	0.02	−0.39***	−0.16*	0.23**
M5	80	No covariates	0.71***	−0.09	−0.49***	−0.31***	0.25**
M5a		Covariates	0.50***	−0.16	−0.40***	−0.27**	0.25**
M6	4T50	No covariates	0.81***	−0.01	−0.49***	−0.25**	0.25**
M6a		Covariates	0.64***	−0.04	−0.39***	−0.22*	0.22**
M7	4T80	No covariates	0.71***	−0.16	−0.49***	−0.40***	0.27**
M7a		Covariates	0.49***	−0.23**	−0.40***	−0.36***	0.25**
M8	SSN50	No covariates	0.82***	0.02	−0.49***	−0.16	0.25**
M8a		Covariates	0.69***	−0.02	−0.39***	−0.12	0.23**
M9	SSN80	No covariates	0.72***	−0.02	−0.49***	−0.22*	0.26**
M9a		Covariates	0.52***	−0.09	−0.39***	−0.18	0.24**

Note 1: Phon = Phonology, Hag = Hagerman. Note 2: Age was a significant covariate in all models for Hagerman, WM, and Speed. PTA was a significant covariate in all models for Hagerman and phonetics. **Bold** indicates significance.

* *p* < 0.05;

** *p* < 0.01;

*** *p* < 0.001.

To investigate if adverse listening conditions (as compared to less adverse) had higher speed and WM paths to Hagerman sentences, models with only a subset of the Hagerman test conditions (4T/SSN/50%/80%) were analyzed separately and in combinations and are reported as model 2–9. Overall, the model fits were similar to the M1a fit in that it was an acceptable fit for most models according to RMSEA, χ^2^/df and CFI. SRMR were slightly higher than the cut-off and TLI were just too low in most models. The slightly worse fit was expected as some information was removed.

Path results of SEM mediation analysis for model 1–9 are presented in Table 4 and loadings of all the SEM models are presented in Supplementary material 1. All paths were in the expected direction and for simplicity the magnitude of the paths will be interpreted (ignoring the minus due to that for some variables, a higher value is better and for others a lower value is better). All models had a strong (0.49–0.69) phonology → Hagerman sentences path, a medium (0.39–0.40) speed → WM path, and a small phonology → speed correlation (0.23–0.25). Even if there is no statistical test that can compare the magnitude of paths from different models, the pattern is that the adverse listening conditions have slightly higher magnitude WM → Hagerman sentences paths than the easier listening conditions (4T = 0.26 > SSN = 0.14, and so on, see Table 3). The speed → Hagerman sentences path was only significant in the most adverse listening condition (M7a) where the outcome was Hagerman test conditions 4T 80. In all models with covariates, age was significantly predictive of Hagerman sentences, processing speed and WM and PTA was significantly predictive of Hagerman sentences and phonology. Effect size indicated good explanatory power of all models.

## 4. Discussion

The current paper has investigated several models with different structural relationships between processing speed, WM, phonology and speech in noise in a group of hearing-impaired individuals. The three main results are, (1) the best fitting model was the model based on the ELU model, (2) the pattern and magnitude of the paths are mostly in line with the ELU model, and (3) models with subsets of the Hagerman test conditions were mostly in line with the prediction that Hagerman sentences in adverse conditions is more predicted by processing speed and WM. These results will be discussed below.

Alternative models did not indicate a good fit of the data according to a number of fit indices. The model providing the least explanatory power was a model of General Speed (Salthouse, 1996; AM1) where processing speed and speed of phonological measures were combined, indicating that speed alone cannot account for performance dB SNR for a given SRT. Additionally accounting for WM in this model (AM2) did not improve the model’s explanatory power of dB SNR for a given SRT. However, a model of General WM/GWM (Baddeley, 2000; AM3), provided the best fit of the data out of the alternative models, indicating the importance of WM and phonology in accounting for dB SNR for a given SRT. Including processing speed in this model did not improve but worsened model fit (AM4). It is worth noting that the difference between AM3 and AM1/2 is not only the inclusion of WM, but that phonology and processing speed was not combined into one construct, suggesting this combination may not be advisable in moving towards a more comprehensible model of cognitive hearing in the present context. The data did also indicate that all three latent variables of interest in our main model (AM5) were significantly predictive of the outcome measure but did not provide a good fit of the data, which can be viewed in support of our main model which uses the same latent constructs but with different relationships, i.e., supporting a possible mediation model.

The ELU model indicated an acceptable fit of the data and a better fit compared to alternative models. Several models using different subsets of Hagerman test conditions were assessed and indicated a pattern of results. Firstly, phonology always contributed to Hagerman sentences, a finding in line with the literature on the ELU model (Rönnberg et al., 2013, 2016). Results showed that worse phonological skills predicted worse Hagerman test scores, or better phonological skills predicted better Hagerman test scores. In addition, older age and more years of hearing aid use is associated with worsened cognitive abilities, which increases the strength of the cognition-dB SNR for a given SRT relationship (Rönnberg et al., 2016). This result may be also in line with a binding concept (where different sources of information are bound to create coherence; Wilhelm et al., 2013) to the extent that different bindings are created in RAMBPHO in the rhyme and gating tasks that are similar to the temporary binding suggested by Wilhelm et al. (rapid updating of temporary bindings occur in WM), the difference lying in the purpose of the models: ELU does not focus on WM as such but how it mediates the communicative outcome of Hagerman matrix sentences.

Secondly, and our third main result, processing speed → Hagerman sentences and WM → Hagerman sentences paths had higher magnitude in the adverse listening conditions. For the processing speed → Hagerman sentences path, it was only significant in the 4T80 condition. The WM → Hagerman sentences paths had consistently slightly higher magnitude in the adverse listening conditions as compared to in the easier listening conditions. Even if these differences are not statistically testable, they follow predictions from the ELU model and the literature highlighting the importance of WM in speech recognition and in particular in adverse listening conditions (Baddeley, 2006; Lunner and Sundewall-Thorén, 2007; Akeroyd, 2008; Rönnberg et al., 2013; Dryden et al., 2017; Strand, 2018). This finding is also in line with the literature on the ELU model in that high WM compensates for adverse listening conditions through the ability to keep representations in mind and thereby compensating for poor phonological skills, while poor WM cannot compensate for adverse listening conditions and a lower amount of speech is understood.

The pattern of our ELU models indicate that the more adverse listening conditions are more strongly associated with WM, both regarding 4T/SSN but also 50/80% where the 50 and 80% conditions showed a significant association with WM, but in the 80% condition relationships were significantly stronger in both significance and coefficients as compared to the 50% condition. Previous findings regarding a difference in the relationship between WM and dB SNR have proposed that the relationship varies in the 50 and 80% conditions. Studies have shown that higher cognitive functioning is crucial in both the 50 and 80% condition (Marsja et al., 2022), while several studies indicate differences between the conditions where a stronger relationship between WM and dB SNR is found in the 80% condition, or only in the 80% condition (Lunner and Sundewall-Thorén, 2007; Larsby et al., 2008, 2011; Stenbäck et al., 2015). The present study supports the former findings to a greater extent.

Moreover, our findings demonstrated that slower processing speed predicted poorer WM which in turn predicted worse Hagerman test scores, or alternatively, better processing speed was associated with better WM which predicted better Hagerman test scores. Or in other words, processing speed was always predictive of WM and the path from processing speed to Hagerman test was only significant in the more adverse conditions (4T) where higher degrees of mismatch can be expected. Considering the ELU model and the notion that it takes time to reconstruct the input in adverse listening conditions, the current findings can be interpreted as highlighting the importance and optimization of processing speed in conditions when WM is activated. This finding is in line with previous findings from the same sample where WM was found to have stronger associations to perception driven sentences (Hagerman sentences) than to context-driven everyday sentences (HINT), thus improving prediction while decreasing demands on postdiction (Rönnberg et al., 2016). Even though processing speed is not a consistent predictor of Hagerman matrix test performance (Akeroyd, 2008), our results showed that good processing speed is necessary for WM to be able to compensate for adverse listening conditions. Thereby, our mediation model highlights the importance of accounting for other possible relationships between variables and not only allowing them to predict the outcome directly.

Generally speaking, the results are also interesting in the sense that a mediation model is more responsive to variability in task demands such that the interplay between the three factors vary dynamically and simultaneously due to differences in these demands (e.g., amount of information that needs to be perceived and recalled). Our mediation model suggests a more nuanced approach to understanding the mechanisms of the ELU system, the consequences of output demands, as well as future comparison with other models where other variables measuring the basic latent concepts also could be employed.

One of the main predictions of the ELU model is that WM is only invoked at mismatch (adverse listening conditions), a prediction that separates the ELU model from some previous models (e.g., TRACE, NAM, and mismatch negativity). Previous studies (e.g., Näätänen et al., 2004) have mainly focused on the importance of physical parameters of mismatch, but not the actual consequences of mismatch-namely that under certain conditions mismatch invokes WM. Our findings, in line with the ELU model, suggest that explicit WM is invoked when a mismatch is large enough. For a review of how the ELU model compares to other models of speech understanding see Rönnberg et al., 2013.

The results of the present study are however somewhat at odds with those by Janse and Andringa (2021). It is possible that the differences between the studies and sample may provide some explanation to this, such as the different measurements used and latent relationships investigated, difference in sample regarding hearing aid use (their sample did not use hearing aid while our did), as well as the use of fast speech, as this can be particularly problematic to hear accurately amongst individuals with hearing impairments (Janse and Ernestus, 2011). In addition, these results may differ from the present study due to the type of masking used. A recent study using similar types of masking as in the present study, SSN and two talker speech (TTS), showed worse performance in the TTS condition as compared to the SSN condition as well as an effect of WM in the TTS condition but not in the SSN condition (McCreery et al., 2020). These results are in line with the present and supports that the more adverse listening conditions are cognitively demanding through the observation that working memory becomes involved in the more adverse tasks but not the less adverse.

Our findings suggest and support the literature in that processing speed is associated with Hagerman sentences (Akeroyd, 2008; Janse and Andringa, 2021), but not in line with other literature suggesting processing speed to be a direct predictor of word recognition (Janse and Newman, 2013; Dryden et al., 2017). Janse and Andringa (2021) findings indicated that processing speed is correlated with word recognition and WM but not directly predictive of word recognition. The findings of the present mediation model may add to the latter study by providing an explanation on how processing speed and WM are associated with dB SNR/word recognition as our model, in line with the latter, indicates that processing speed is not a direct predictor of Hagerman sentences but instead is mediated through WM. It should be noted that the present and latter study (Janse and Andringa, 2021) did not use the same structure of word recognition-the latter study focused on isolated words whereas the present on 5-word sentences. It is, therefore, possible that the present mediation model is applicable to both structures, however, future research is needed to confirm this.

### 4.1. Limitations

The comparison between models is somewhat biased in that the n200 dataset was designed to test parameters of the ELU model and by that might not have the ideal test for some of the alternative models. It is important to note that the present study is only performed on individuals with hearing aid data. This affects the generalization of results and future studies should investigate if the results hold for individuals without hearing aids and/or without hearing loss.

### 4.2. Conclusion

In the present study, we modelled the structural relationships of WM, processing speed, phonology and Hagerman sentences in a group of hearing-impaired individuals. Results indicated that phonology was predictive of Hagerman sentences in all our models and processing speed was always predictive of WM. The path from processing speed to WM to Hagerman sentences was only significant in the more adverse conditions (Hagerman test condition 4T). Results were in line with the predictions of the ELU model and supported that WM is invoked to compensate for adverse listening conditions and is *only* invoked in the more adverse listening conditions. In addition, the results highlight the importance and role of processing speed in relation to WM during adverse listening conditions.

## Data availability statement

The original contributions presented in the study are included in the article/Supplementary material, further inquiries can be directed to the corresponding author/s.

## Ethics statement

The studies involving human participants were reviewed and approved by Etikprövningsmyndigheten. The patients/participants provided their written informed consent to participate in this study.

## Author contributions

LH, HD, and JR conceptualized the contributions and reviewed the paper and provided critical revisions of the manuscript. LH performed the analysis and wrote the manuscript. All authors contributed to the article and approved the submitted version.

## Funding

This work was supported by grant no. 2017-06092 (Anders Fridberger) and the Linnaeus Centre HEAD grant no. 349-2007-8654 (JR) both from the Swedish Research Council.

## Conflict of interest

The authors declare that the research was conducted in the absence of any commercial or financial relationships that could be construed as a potential conflict of interest.

## Publisher’s note

All claims expressed in this article are solely those of the authors and do not necessarily represent those of their affiliated organizations, or those of the publisher, the editors and the reviewers. Any product that may be evaluated in this article, or claim that may be made by its manufacturer, is not guaranteed or endorsed by the publisher.
