# Supplementary material for: A structural equation mediation model captures the predictions amongst the parameters of the ease of language understanding model
Source: Front Psychol. 2023 Mar 3;14:1015227. doi: 10.3389/fpsyg.2023.1015227 (PMC10020708; doi:10.3389/fpsyg.2023.1015227)
Supplement: Supplementary file 1 [file Table_1.docx]

Supplementary Material: Homman et al., 2023

| Table 1 Supplementary material: Loadings of all models including covariates | | | | | | | |  |  |  |
| --- | --- | --- | --- | --- | --- | --- | --- | --- | --- | --- |
| Latent variable | | Model |  |  |  |  |  |  |  |  |
|  |  | M1a | M2a | M3a | M4a | M5a | M6a | M7a | M8a | M9a |
| Speed | PM | .36*** | .37*** | .36*** | .36*** | .37*** | .36*** | .37*** | .36*** | .37*** |
|  | LD | .53*** | .54*** | .53*** | .54*** | .52*** | .54*** | .54*** | .53*** | .52*** |
| WM | RST | .61*** | .61*** | .62*** | .62*** | .60*** | .62*** | .59*** | .62*** | .61*** |
|  | SWPST | .55*** | .55*** | .55*** | .55*** | .54*** | .55*** | .54*** | .55*** | .55*** |
|  | VSWM | .68*** | .69*** | .68*** | .67*** | .70*** | .67*** | .71*** | .67*** | .68*** |
| Phonology | Rhyme | .15* | .15* | .15* | .15* | .16* | .15* | .16* | .15* | .16* |
|  | GAC | .81*** | .82*** | .81*** | .80*** | .83*** | .80*** | .84*** | .81*** | .82*** |
|  | GAvC | .85*** | .84*** | .85*** | .85*** | .83*** | .85*** | .82*** | .85*** | .84*** |
|  | GAV | .67*** | .67*** | .67*** | .68*** | .66*** | .67*** | .66*** | .68*** | .66*** |
|  | GAvV | .67*** | .67*** | .67*** | .67*** | .67*** | .67*** | .66*** | .67*** | .67*** |
| Hagerman | FasSSN50 | .88*** | - | .89*** | .89*** | - | - | - | .88*** | - |
|  | FasSSN80 | .77*** | - | .75*** | - | .73*** | - | - | - | .74*** |
|  | Fas4T50 | .80*** | .78*** | - | .79*** | - | .79*** | - | - | - |
|  | Fas4T80 | .68*** | .67*** | - | - | .72*** | - | .70*** | - | - |
|  | NPSSN50 | .88*** | - | .90*** | .90*** | - | -- | - | .92*** | - |
|  | NPSSN80 | .71*** | - | .70*** | - | .73*** | - | - | - | .77*** |
|  | NP4T50 | .83*** | .80*** | - | .85*** | - | .81*** | - | - | - |
|  | NP4T80 | .80*** | .80*** | - | - | .81*** | - | .80*** | - | - |
|  | NRSSN50 | .81*** | - | .81*** | .83*** | - | - | - | .82*** | - |
|  | NRSSN80 | .73*** | - | .71*** | - | .73*** | - | - | - | .67*** |
|  | NR4T50 | .85*** | .86*** | - | .87*** | - | .89*** | - | - | - |
|  | NR4T80 | .60*** | .63*** | - | - | .66*** | - | .64*** | - | - |
| Covariates | |  |  |  |  |  |  |  |  |  |
| PTA | Speed | .14 | .13 | .14 | .14 | .14 | .14 | .14 | .14 | .14 |
|  | WM | -.01 | -.01 | -.01 | -.01 | -.01 | -.01 | -.01 | -.01 | -.01 |
|  | Phonology | .18* | .18* | .18* | .18* | .18* | .18* | .18* | .18* | .18* |
|  | Hagerman | .30*** | .35*** | .25*** | .27*** | .35*** | .31*** | .39*** | .23*** | .32*** |
| Age | Speed | .36*** | .36*** | .36*** | .36*** | .36*** | .36*** | .36*** | .36*** | .36*** |
|  | WM | -.23* | -.23* | -.23* | -.23* | -.22* | -.23* | -.22* | -.23*** | -.23*** |
|  | Phonology | .09 | .09 | .09 | .09 | .10 | .10 | .10 | .09 | .09 |
|  | Hagerman | .16* | .16* | .17* | .14* | .21** | .13* | .21** | .16* | .21** |
| *p<.05; **p<.01; ***p<.001 | | |  |  |  |  |  |  |  |  |
| **Note: As all the models including covariates indicated a better fit of the data, only these models are reported here | | | | | | | | | | |
